# Supplementary material for: Species specific morphological alterations in liver tissue after biliary occlusion in rat and mouse: Similar but different
Source: PLoS One. 2022 Jul 26;17(7):e0271975. doi: 10.1371/journal.pone.0271975 (PMC9321426; doi:10.1371/journal.pone.0271975)
Supplement: Attachment — Submitted filename: response to reviewers.docx [file pone.0271975.s001.docx]

5. Review Comments to the Author

Please use the space provided to explain your answers to the questions above. You may also include additional comments for the author, including concerns about dual publication, research ethics, or publication ethics. (Please upload your review as an attachment if it exceeds 20,000 characters)

Reviewer #1: Overall, the article reads well and contains good clear data to support the conclusions. The conclusions themselves are not overly exciting, but may be of practical use to researchers in the field. The one concern I have is whether strain-specific differences may exist, which is not explored in this study. Perhaps the authors should comment more on this in the discussion.

A major point is the word choice. There are several instances where poor word choice makes the resulting statement unclear. Specifically:
- In the abstract, the phrase “only tiny bile infarcts than mice” is grammatically incorrect. Use “only tiny bile infarcts compared to mice” instead.
- In the introduction, the phrase “rational… reasons” is redundant. Do you mean practical? Also, it is not clear what you mean by “for the abundance of reagents”. Are you trying to say that mice require lower volumes or amounts of reagents
- In results, rephrase "albumin as parameters" to "serum albumin as a parameter". In the legend of Figure 1 where you say the mice did not "overreach" their weights, change it to "exceed". This is a better choice of words. Likewise, hepatocyte "compartment" is an inaccurate term. Use "hepatocyte mass" or "hepatocellular component" instead

We thank you very much for your important comments and references. We changed the wording and screened the manuscript for unnecessary or misleading description. We do very appreciate your helping suggestions.

A second major point is the lack of statistical comparison between groups. It is important to know if the observed differences are statistically significant. You should be using non-parametric tests based on your numbers.

We included statistical comparison of the groups. We marked the significances differences in the tables and figures.

A few minor points:
- I don't think that 100% vs. 92% survival is really a substantial difference, especially since it is much higher than other studies are reporting.

We agree with you that the difference between the survival rates is not very impressive. Since only one operative intervention (the biliary occlusion) led to a different mortality in rats and mice, we do think that this small numeric difference makes and describes a strong species specific distinction. Today we cannot explain these different survival rates.

- It is unnecessary and somewhat distracting to bold and underling mice and rats.

We want to apologize for the distracting character of the visual marking of rats and mice within the description of the results. By using the visual marking, we wanted to simplify the distinction between rats and mice. We have withdrawn this labelling according to your recommendations.

- You don't need to put quotes around "biliary infarcts" every time, since it's a term you chosen to use.

We apologize for using to many “quotations”, we have eliminated these and changed the writing according to your recommendations.

- It's not clear what is meant by "portal fields" and "biliary proliferates". Do you mean periportal? Cholangiocyte proliferation?

We apologize for the non-intended confusion by our wording. We do distinct between the ductular reaction in the periportal area including the portal fields/tract and the biliary proliferates in the hepatocellular compartment as description for cholangiocytes` proliferation due to ductular reaction at two different locations in the liver architecture. We use these different terms in order to simplify the differentiation between both intrahepatic localisation of cholangiocytes` proliferation (CC). Therefore, we use portal fields as “abbreviation” for the periportal CC and biliary proliferates in the hepatocellular compartment. We did delete the term extraportal in the manuscript.

Reviewer #2: This study aimed to demonstrate the suitability of using rats or mice for experimental occlusive cholestasis. Authors show that the main difference between both species are the severity and kinetics of histological alterations, and propose that septic issues are the most important factors impacting in mice survival. Although the finding might be of relevance, several issues still need to addressed before further steps.

The presentation of the manuscript is inadequate, authors repeat the figure legends in the manuscript and in the figures section; the words: rats and mice should not be either highlighted or underlined. Authors excessively use parenthesis throughout the manuscript; for example, in the abstract, conclusion section is too confused since it is not fluently written. Authors should avoid parenthesis and instead, they should rewrite the conclusion. In introduction: Mostly, rodents (rat, mouse) were used, because of rational and scientific reasons. It should be: Mostly, rodents either rats or mice HAVE BEEN used, because of rational and scientific reasons.

We do appreciate your important recommendations. We have changed the manuscript according to your suggestions. We have marked all changes with yellow.

What does stand for 28“d” in the following sentence? At five time points (1, 3, 7, 14, 28d, n=5/time point) after tBDT,

We used the international abbreviation d for days. We changed the sentence and the description for time intervals according to your recommendations.

According to The International System of Units (SI), hours and other units must be indicated as symbol (h), not as abbreviation (hrs); moreover, they should be separated from number; for example: 0.005 mg/kg instead of 0.005mg/kg, and so on and on. Authors should carefully review the whole manuscript. Supplementary Tables were not included in the manuscript or review.
The manuscript contains a number of grammar and typo issues that need to be carefully reviewed; for example, the following sentence in result section, at page 6, states: “The rats showed AN maximal weight loss…”; but it must be: “The rats showed A maximal weight loss…”.

We want to thank you very much for your important comments. We changed the manuscript and the writing according to your recommendations. We did upload the tables within the submission procedure. However, we included the tables in the revised manuscript and additionally submitted the tables in extra files as the tables demand extra space due to their formatting. We are very sorry that you have had no access to the single data of our study.

All abbreviations shown in figure 2 must be defined in the legend of the figure. For example, what does stand for CC, Hep, etc?

We apologize for the missing explanation of the abbreviations we used. We have included the explanations as you recommended. (CC stands for cholangiocellular, Hep for hepatocellular).

Figure legends contain excessive explanation which should be included in result section. Figure legends should contain a brief description about what images are showing but not a full explanation.

We apologize for the non-intended redundance. We shortened the figure legends according to your comments.
