# Supplementary material for: Species specific morphological alterations in liver tissue after biliary occlusion in rat and mouse: Similar but different
Source: PLoS One. 2022 Jul 26;17(7):e0271975. doi: 10.1371/journal.pone.0271975 (PMC9321426; doi:10.1371/journal.pone.0271975)
Supplement: Attachment — Submitted filename: response to reviewers_PONE-D-21-36588R2.docx [file pone.0271975.s002.docx]

**Reviewer #1**: Thank you for taking the time to address my comments. I still feel, however, that there are several issues that need to be addressed before publication.

Overall, there are still several instances of poor sentence structure and unusual or incorrect word choice. For instance, the sentence fragment "Moreover, transgenic mice strains facilitating further comparative molecular research." in the Introduction and the use of 'kinetic' as a noun throughout. I would recommend review by a native English speaker.

Section specific comments:
1. Abstract
- include p values

We have included the p-values in the abstract. Since we determined the dedicated values at five different time points, we mention only the significant values and their p-value. By doing so we want to avoid confusion between the values and to facilitate understanding of the data. We agree with you that a p-value below 0.07 is not associated with a “stronger” significance. Therefore, we distinct only between the p-value below or above/equal 0.05.

- write all abbreviations in full upon their first use (do not include an abbreviation if the word is only used once)
We agree to use abbreviations after the word was written in full. We changed the manuscript.

2. Methods
- state whether assessment was done in a blinded fashion

We thank you for your interesting recommendation. We agree with you that a blinded assessment of data assures an objective evaluation of information. In particular in clinical studies the double-blinded assessment is an obligatory part of the study design. We agree with you that the clinical assessment of animals in experimental projects cannot be done in a blinded fashion. The histological slides were predominantly quantified by a software (batch analysis). However, the training of the software required always a human control. The blood test were analysed by an automated chemical device. However, the results were checked always for their validity by a human who was informed at least about time points, mostly also about the species. Since such quality controls in experimental projects are performed at regularly basis in our laboratory, we cannot state that the assessment was done in a blinded fashion.

3. Results
- include p values in the text

We have included the p-values in the results.

- include data for rats and mice in the same tables, since you are making a comparison between them

We thank you for your important recommendation. We decided to present the data for rats and mice in separate tables facing diverse considerations: I.) we want to avoid confusion between data of rats and mice; II.) we want to facilitate comprehension and comparability of the huge amount of data. Therefore, we like to adhere to the presentation of the data of rats and mice in separate tables.

- the appropriate abbreviations for alanine aminotransferase and aspartate aminotransferase are ALT and AST, respectively

We thank you for your interesting request. We agree with you, that ALT and ALAT are equivalent abbreviations for alanine aminotransferase (as well as AST and ASAT are for aspartate aminotransferase) in the international literature. We have used ALAT and ASAT as abbreviations in numerous publications, and until now no international or national journal has ever asked for a correction into AST or ALT, respectively. We apologize for our decision of further use of ASAT and ALAT as abbreviations.

- report the p value for mortality
- by my calculations it was 0.0740, which is generally not considered significant
- in light of this, you should report this a trend toward increased mortality in mice

We agree with you that the differences in the survival rate were not significant with a p=0.070484. We included this value in the results section. In addition we included the description of significant differences.

- do you have baseline values for laboratory chemistry and weight (i.e., from untreated animals)?
- if so you should report these

Unfortunately, we do not have baseline values of untreated rats and mice on our time points of interests.

- I have several problems with table 6 and I think it is best removed, all things considered
- the human column contains little useful information since you report most of the characteristics are dependent on the underlying disease state

We agree with you that a comparison of results between different species is still challenging. By including Table 6 we want to facilitate and strengthen the understanding of important issues: I.) The differences in size (e.g., body weight, organ weight) and the differences in progression of weight/size due to tBDT (e.g., also the missing data in human in relation to the current literature); II.) the anatomical differences beyond the size issue (e.g., presence of a gall bladder, lobulation of liver); III.) the differences in stress resistance and susceptibility to complications (e.g., septic conditions, blood loss, anaesthesia, surgery); IV.) brief description of the characteristics/differences of the hepatobiliary remodelling after tBDT; V.) the massive differences in knowledge about the genetical background/ opportunities of genetical modification in relation to specific topics of the experimental cholestasis research. We included a column with human data from the literature to illustrate the differences in knowledge and the possible impossible comparison with human data (e.g., clinical studies, textbook data). We agree with you that in this case, the understanding of such a concentrated comparison of seven issues is best supported by the tablet format.

- furthermore, the indication that 'genetical models' of humans are 'not applicable yet' is bizarre, as it would be highly unethical to ever create transgenic humans for research

We agree with your statement that it is unethical to create genetically altered humans for research. Obviously only you misinterpreted our sentence. We have changed the text in this line of the table 6. However, in medicine (e.g., cancer research, immunologic diseases research) we are about to gain more insights in the epigenetic background in animals (and humans) enabling or inhibiting or avoiding cancer and immunologic diseases. In experimental research, with the establishment of genetically modified species (predominantly mice; to a lesser extent rats) the scientific community built a network providing growing knowledge of altered pathways in relation to the genetical modification and diseases.

- ratings in categories 'Tolerance to surgical stress' and 'Susceptibility to complications' appear highly subjective and it is unclear how you distinguish between 'low', 'moderate', and 'high. figure 6 provides all of the relevant information in this table, if you think it is important to compare anatomical difference I would do so in a figure

We agree with you that rating categories are at risk for smooth transition zones. However, in relation to our categories low vs. moderate vs. high concerning tolerance to surgical stress and susceptibility to complications we referred to the data of survival, body weight gain and stress score as already described in the section material and methods and section results. We included a description in the legend of the Table 6.

We agree with you that the Figure 6 presents the comparison of the differences in basic characteristic parameters (e.g., stress resistance/survival, characteristics of hepato-biliary remodelling after tBDT) for selection of the appropriate species (rat vs. mouse) in cholestasis research. Again, we agree with you that Table 6 and Figure 6 are complementary presentations of the results of this study and do support the fast understanding of species` specific differences.

4. Discussion
- conclusions are overstated
- the findings don't 'confirm' that rats are the best choice for surgical models
- they provide researchers with considerations when choosing between one or the other
- mice could still be used for surgical models, but researchers should understand that they are more susceptible to injury
- discuss limitations of your study

We thank you for your interesting recommendations. We included a paragraph with discussion of the limitations of our study and changed the sentences according to your recommendations.

The biggest factor that limits the applicability of your findings is the use of only male animals
- it seems that the impact of sex would be an important consideration when choosing an animal model for researchers in this field

We agree with you that the (still used) limitation on the male sex in experimental research maybe does harbour the risk of misleading data/ results. Since until now (nearly) all scientific data in cholestasis research raised from male animals, we decided to focus on male animals. We discussed this problem within the “limitations paragraph” in the discussion section.

- another is the unknown impact of strain on biliary injury
- it's hard to generalize these findings when you've only looked at one strain of each

We agree with you that every limitation of a study design harbour the risk of challenging results. In order to avoid a never ending project, we used only the male animals of one strain without genetical modifications per species and limited the study design on three endpoints. The strains were inbred C57BL/6N mice and inbred Lewis rats [2,28,34]. The endpoints were survival, stress resilience (e.g., body weight gain, stress score) and the hepato-biliary remodelling after tBDT. We used the mostly used strain of either species and only the male animals according to the current literature. Furthermore, we have not found equivalent genetically altered strains of either species in cholestasis research that could be used for a detailed evaluation, especially to define species` specific differences in hepatobiliary remodelling after tBDT. Therefore, we refrained from including genetically modified strains of either species.

**Reviewer #2:** The authors have properly addressed my suggestions.
I believe that the corrected version of the manuscript is now acceptable for further steps in the publication process by Plos One journal.

We thank you very much for your constructive comments.
